# Dissection of the amyloid formation pathway in AL amyloidosis

**DOI:** 10.1038/s41467-021-26845-0

**Published:** 2021-11-11

**Authors:** Pamina Kazman, Ramona M. Absmeier, Harald Engelhardt, Johannes Buchner

**Affiliations:** 1 Department Chemie, Technische Universität München, 85748 Garching, Germany; 2 Department Molecular Structural Biology, Max-Planck-Institute of Biochemistry, 82152 Martinsried, Germany

**Keywords:** Biophysical chemistry, Protein aggregation

## Abstract

In antibody light chain (AL) amyloidosis, overproduced light chain (LC) fragments accumulate as fibrils in organs and tissues of patients. In vitro, AL fibril formation is a slow process, characterized by a pronounced lag phase. The events occurring during this lag phase are largely unknown. We have dissected the lag phase of a patient-derived LC truncation and identified structural transitions that precede fibril formation. The process starts with partial unfolding of the V_L_ domain and the formation of small amounts of dimers. This is a prerequisite for the formation of an ensemble of oligomers, which are the precursors of fibrils. During oligomerization, the hydrophobic core of the LC domain rearranges which leads to changes in solvent accessibility and rigidity. Structural transitions from an anti-parallel to a parallel β-sheet secondary structure occur in the oligomers prior to amyloid formation. Together, our results reveal a rate-limiting multi-step mechanism of structural transitions prior to fibril formation in AL amyloidosis, which offers, in the long run, opportunities for therapeutic intervention.

## Abstract (web summary)

AL amyloidosis is caused by the accumulation of overproduced light chain (LC) fragments as fibrils in patient organs and it is the most prevalent systemic amyloidosis. Here, the authors combine biochemical and biophysical experiments to characterise the lag phase of a patient-derived truncated LC and they identify structural transitions that precede fibril formation.

## Introduction

Light chain (AL) amyloidosis is the most common type of systemic amyloidosis^1^. The disease is caused by an underlying plasma cell dyscrasia that entails the elevated expression and secretion of free antibody light chains (LC)^2,3^. Patient-specific mutations in the LC, which destabilize the native fold and consequently trigger fibril formation are an important element of the disease^4–7^. Furthermore, the circulating free LCs often undergo proteolytic cleavage prior to assembly into insoluble amyloid fibrils^8–11^. N-terminal fragments, comprising mostly the variable domain (V_L_) of the LC often represent the amyloidogenic species. The hallmark of AL amyloidosis is the transformation of soluble monomeric protein into insoluble amyloid fibrils^12,13^. Their presence correlates strongly with the disease and the impairment of organs in which they are deposited. Structurally, substantial rearrangements of the V_L_ domain in AL fibrils compared to the native fold have occurred as resolved by cryo-EM. During restructuring, the internal disulfide bond is retained and hydrophobic core residues become surface-exposed^14,15^. Like in other amyloid diseases, the growth phase of AL fibrils is preceded by a comparably long and rate-limiting lag phase in vitro^16–18^. So far, little is known about the structural events taking place during this phase. For different amyloid diseases, oligomeric intermediates of the amyloidogenic proteins and a nucleated polymerization mechanism have been suggested^16,19–22^. Capturing these species remains challenging, due to their transient appearance in low concentrations and the high energy states of the specific intermediates^16,18^. In AL amyloidosis, the presence of oligomers forming during the lag phase and the accompanied structural transitions prior to amyloid formation have not been investigated so far. Understanding the pathway and molecular mechanism of reactions preceding fibril formation of pathogenic LCs is important to identify potential therapeutic intervention points at early stages of the disease. Here, we elucidated the processes taking place during the lag phase prior to fibril formation of the well-studied pathogenic V_L_ domain Pat-1^6^. We identified intermediate oligomeric species on the fibril pathway and associated structural rearrangements using a broad range of biophysical analyses.

## Results

### Fibril formation is preceded by the disappearance of soluble V_L_ monomers

**Fig. 1:**
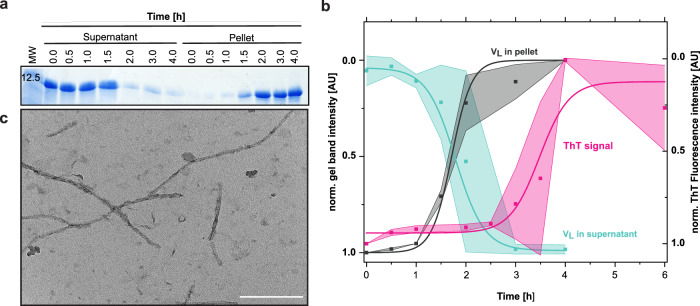
Transition from soluble V_L_ Pat-1 to insoluble fibrils. **a** Soluble and insoluble fractions of Pat-1 V_L_ during incubation at amyloid-promoting conditions. Samples were taken at the timepoints indicated and run on an 18% SDS-gel. **b** Quantified and normalized band intensities of the SDS-gel are shown in turquoise for the soluble V_L_ domain (t_1/2_ = 1.79 h) and in black for the insoluble fraction (t_1/2_ = 1.71 h). Shades represent the SEM of *n* = 2. Fibril formation of Pat-1 V_L_ as monitored by ThT fluorescence is shown in pink (t_1/2_ = 3.48 h). Values were fit to a Boltzmann function Shades represent the SEM of *n* = 3. **c** TEM micrograph of fibrils formed after 3 h of incubation. The scale bar represents 200 nm.

In AL amyloidosis, fibril formation in vitro is usually preceded by a long lag phase after which fibrils form in a rapid and cooperative reaction as monitored by ThT fluorescence^16,23^. The molecular events occurring in this lag phase are still elusive. For a better understanding of AL amyloidosis, we set out to resolve the molecular mechanism of structural changes occurring during the lag phase for the well-characterized patient LC truncation Pat-1 that represents the major component of the deposited fibrils. The structure and amyloidogenic properties of Pat-1 had been reported previously^6^. In this study, two disease-causing mutations (L15P L82Q) had been identified in Pat-1 compared to the germline sequence, which did not form fibrils. For the lag phase analysis, we used the Pat-1 V_L_, the double mutant Pat-1 L15P L82Q and the respective germline sequence WT-1. To obtain insights into the molecular events occurring during the lag phase, we first monitored changes in the solubility of the V_L_ domain under conditions favoring rapid fibril formation (37 °C, shaking at 750 rpm, 0.5 mM SDS). Low concentrations of SDS are typically used as a destabilizing agent to accelerate fibril formation of amyloid proteins^6,24^. Aliquots were taken at different timepoints during incubation and separated into soluble and insoluble fractions by centrifugation. Analysis of the fractions by SDS-PAGE and quantification of the bands showed that during the first hour of incubation, the amount of soluble V_L_ domain did not change significantly (Fig. 1a). Then, a decrease of soluble Pat-1 and a concomitant increase of Pat-1 in the insoluble fraction were observed over time. The half times of the reactions were very similar, with a t_1/2_ of 1.79 h ± 0.11 for the decrease of the soluble form and t_1/2_ of 1.71 h ± 0.30 h for the increase in the insoluble species (Fig. 1b). After 2 h, almost no protein was left in the soluble fraction. Fibril formation was confirmed by the measurement of the ThT fluorescence. Fibrils started to form after 2 h and the half time of the reaction was 3.5 h (Fig. 1b). After 4 h, no further increase in ThT fluorescence was visible, thus no further fibril growth seems to take place while ongoing structural rearrangement cannot be excluded. The presence of fibrils was confirmed by TEM micrographs (Fig. 1c). For Pat-1 L15P L82Q, fibril formation occurred even faster while the germline did not form fibrils as confirmed by ThT fluorescence and TEM (Supplementary Figs. 1, 4d, h). When we transferred the Pat-1 at different timepoints during the lag phase to lower temperatures (4 °C or 20 °C) and stopped shaking, ThT fluorescence analysis showed that no further fibril formation occured over a time period of 3 h (Supplementary Fig. 2). This allowed us to dissect the lag phase and perform detailed analyses of the samples.

### Different oligomeric intermediates are part of the pathway to fibril formation

**Fig. 2:**
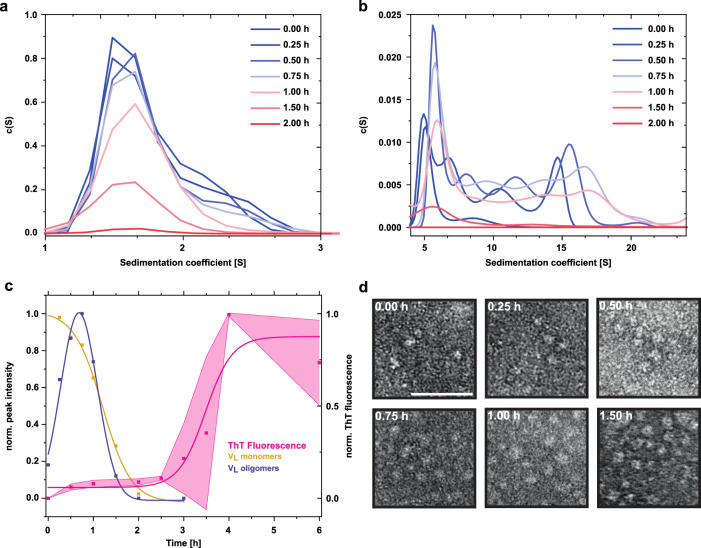
Oligomeric species present in the lag phase of the amyloid pathway of Pat-1 V_L_. AUC sedimentation profiles over time of **a** monomeric and dimeric species and **b** oligomeric species. **c** Normalized AUC peak quantification of monomeric (yellow) and oligomeric (purple) species during the lag phase of fibril formation. The data for monomeric species were fit to a Boltzmann function, the data for oligomeric species were fit to a Gaussian function. Shade of the ThT Fluorescence represents the SEM of *n* = 3. **d** Section of TEM micrographs shown in Fig. S5 of the oligomer and fibril formation over time. Scale bar represents 50 nm.

Due to the denaturing conditions, SDS-PAGE analysis does not provide information on noncovalent structural alterations, specifically oligomerization, taking place during the lag phase. Thus, to determine potential changes in quaternary structure, we performed analytical ultracentrifugation (AUC). For a better resolution of the different species we increased the protein concentration. The sedimentation analysis revealed that at the start point (0 h), Pat-1 was present mainly as a monomer with a small fraction of dimers (Fig. 2a, Supplementary Fig. 3). After 15 min incubation, a range of oligomers became visible as four distinct peaks in the range from 4 to 20 S in addition (Fig. 2b, Supplementary Fig. 3). The amount of oligomeric species increased during the first 30 min, while the monomer fraction decreased. After 45 min, the oligomeric species reached a peak, while about half of the sample was still monomeric. After that timepoint, both oligomers and monomers decreased and disappeared completely, and fibrils evolved (Fig. 2a–c). At each timepoint tested, the concentration of oligomers was lower than the concentration of monomers, as determined by the area under the curves (Fig. 2a–c). For Pat-1 L15P L82Q, the appearance of oligomers before fibril formation was also observed (Supplementary Fig. 4a, b). Since the monomer peak (1.5 S) shows a shoulder towards higher S values (2.1 S) from the beginning of the incubation onwards, we conclude that initially V_L_ dimers form in Pat-1 and Pat-1 L15P L82Q (Fig. 2a, Supplementary Figs. 2, 4a-c). These dimers remain present in low amounts during the first hour of incubation. After that, they cannot be detected unambiguously due to the lower concentration of monomers and the lack of resolution provided by the fitting software. TEM micrographs confirmed the presence of oligomeric species after 15 min incubation of Pat-1 and immediately before fibril formation after 1.75 h in Pat-1 L15P L82Q. Over time, the oligomer amount increases and fibrils for Pat-1 could be detected after 3 h and after 2 h for Pat-1 L15P L82Q consistent with the results of the ThT assay (Fig. 2d, Supplementary Figs. 1, 4d, 5). Large oligomers were visible in the TEM micrographs in a lower number, which could be due to their dissociation under the acidic conditions during negative staining. The germline formed oligomeric species with a clustering morphology. However, here no fibrils but amorphous aggregates were formed (Supplementary Fig. 4e-h).

### The V_L_ secondary structure changes during oligomerization

**Fig. 3:**
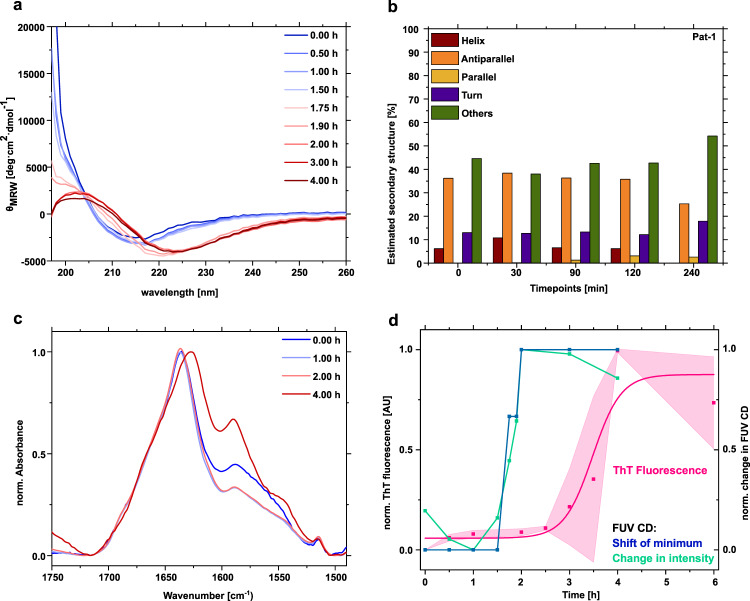
Secondary structure changes during oligomer formation monitored by FUV CD-and FTIR spectroscopy. **a** Comparison of changes in the secondary structure of Pat-1 in the lag phase. The spectra show the average of 10 individual scans. **b** Secondary structure analysis of the CD spectra of Pat-1 using the BeStSel algorithm. **c** Secondary structure changes in the lag phase monitored by ATR-FTIR after H/D exchange. The spectra of the amide I and II region were scaled to an absorbance of 1 and baseline corrected. **d** Comparison of the shift of the minimum in the spectra of different timepoints during the lag phase (blue), the change of intensity at 210 nm (green) with the fibril formation kinetics followed by ThT fluorescence (pink). Shades of the ThT fluorescence represent the SEM of *n* = 3.

As the native V_L_ domain and AL fibrils differ substantially in side chain interactions^14,15^, conformational remodeling of V_L_ has to occur in the lag phase. To detect potential secondary structure changes, we followed the reaction over time by far UV (FUV) circular dichroism (CD) spectroscopy. At the start of the reaction, Pat-1 V_L_ showed a minimum at 218 nm followed by an amplitude as expected for the antiparallel β-sheet native structure of Ig domains (Fig. 3a)^25^. After 0.5 h incubation, the amplitude of the minimum at 218 nm increased. The alterations observed during the lag phase indicate a reorientation and partial unfolding of secondary structure elements. This trend continued further during 1.5 h of incubation. After 1.75 h, the CD minimum shifted to 220 nm and the amplitude increased. At the same time, a maximum at 204 nm emerged. The shift of the minimum and the simultaneous intersection of the *x*-axis at higher wavelengths indicates the formation of parallel β-sheets^26^. Thus, this timepoint marks an important conformational rearrangement. During the lag phase, the high voltage (HT) at the detector, which reflects the absorption of light by the sample, also changed. The signal decreased during the lag phase, which means more light reaches the detector (Fig. S6). This in turn suggests that structural changes affecting the signal take place. After 2 h, the minimum shifted further to 224 nm and the maximum at 204 nm was more pronounced. This maximum is indicative of supramolecular β-sheet-rich amyloid structures^27^. A similar effect was also observed for Pat-1 L15P L82Q but not for the germline WT-1 (Supplementary Fig. 7a). Analysis of the CD data with the BeStSel algorithm^28^ revealed an decrease of antiparallel β-strands with a slight increase in parallel β-strands and the unfolded fraction for Pat-1 and Pat-1 L15P L82Q but not for WT-1 (Fig. 3b, Supplementary Fig. 7b). The shift of the minimum in the FUV CD spectra over time precedes the appearance of fibrils, as the ThT fluorescence starts to increase after the wavelength shift of the CD minimum to 224 nm (Fig. 3d). Interestingly, even before the shift in the CD minimum occurs, the signal of the amplitude at lower wavelengths decreases in the first hour, which might indicate partial unfolding. The changes are particularly noticeable at a wavelength of 210 nm, which is why these were used for comparison in Fig. 3d. The following increase of the amplitude between 1 and 2 h of the lag phase seems to correspond to domain rearrangements during oligomer formation and rearrangement. Upon fibril formation, the signal remains constant. Taken together, the CD analysis suggests that despite the apparent rearrangement of secondary structure, secondary structure elements remain present during the lag phase. Concomitant with fibril formation, the CD data suggest a structural rearrangement event leading from antiparallel to cross β-sheets.

To further analyze secondary structure changes during the lag phase we used attenuated total reflection Fourier transform infrared spectroscopy (ATR-FTIR). In order to minimize contributions in the Amid I region originating from residual water bound to buffer components we recorded spectra after H/D exchange (Supplementary Fig. 7c). The overall content of β-structure remains unchanged in samples of mono- and oligomers while fibril formation results in a peak shift from 1638 to 1625 cm^−1^ because of intermolecular β-sheet stacking in the fibrillar structure (Fig. 3c)^29^. Concomitantly, the shoulder around 1685 cm^−1^ disappears. This observation indicates a decrease of antiparallel β-sheet content^30^. The peaks at 1590 and 1515 cm^−1^ mainly belong to side chain absorptions and Tyr, respectively^31^.

### The V_L_ domain structure changes during oligomerization

**Fig. 4:**
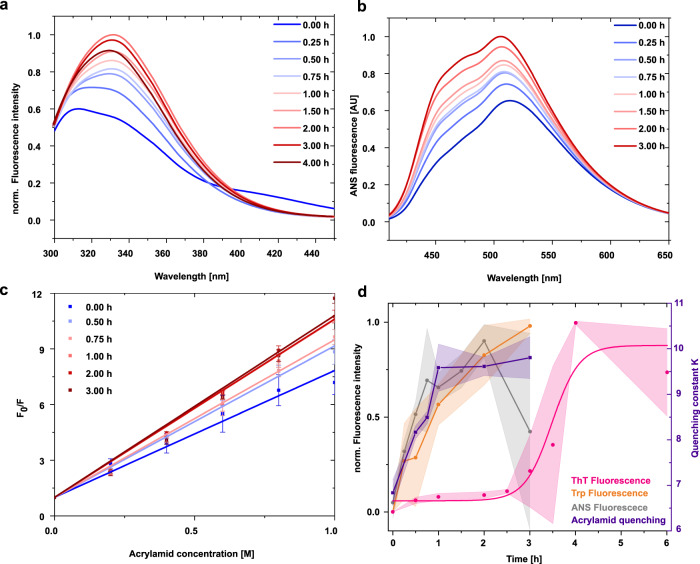
Structural changes of Pat-1 during the lag phase. **a** Change of tryptophan fluorescence. Each emission spectrum is the average of three individual scans. **b** Change of surface hydrophobicity followed by ANS fluorescence. **c** Change of tryptophan fluorescence upon quenching with 0–1 M acrylamide. The curves show the respective F_0_/F values of the Stern-Volmer equation, Error bars represent the SEM of *n* = 3. **d** Comparison of the normalized change of ANS fluorescence (gray), Trp exposure (orange) and the quenching constant K of the acrylamide quenching calculated by Eq. (1) (purple) in comparison to changes in the ThT signal (pink). Shades represent the SEM of *n* = 3.

To monitor structural changes in the core of the V_L_ domain, we analyzed the fluorescence of the single intrinsic tryptophan as a sensitive and specific probe since its emission intensity is quenched in the native state by the disulfide bridge located in the core of the β-barrel and becomes higher upon unfolding^25^. Thus, conformational changes involving the protein core can be monitored by a change in the fluorescence amplitude of tryptophan. When we analyzed changes in the fluorescence emission during the lag phase, we observed a steady increase from the beginning onwards indicating that structural rearrangements take place in the protein core (Fig. 4a). The fluorescence intensity reached its maximum after 3 h, at the same time when fibrils have formed (Fig. 4d). Pat-1 L15P L82Q and WT-1 also showed an increase in tryptophan fluorescence which occurred rapidly upon fibril formation for the double mutant while the germline shows no fibril formation (Supplementary Figs. 4, 8a, b).

To further probe structural changes of the domain surface, we used the fluorophore ANS which binds specifically to surface-exposed hydrophobic patches in a protein. Upon binding, the fluorescence intensity increases and the emission maximum shifts to lower wavelengths^32^. Following ANS fluorescence during the lag phase, we observed an increase in ANS fluorescence intensity from the beginning of the incubation until a maximum was reached after 2 h. The increase in surface hydrophobicity occurs for both, Pat-1 L15P L82Q and the germline, although to a much greater extent in the double mutant (Supplementary Figs. 8c, d). Since the germline also forms oligomers and aggregates, an increase in ANS binding is reasonable. Thus, our results indicate that rearrangements in the environment of the tryptophan in the core also leads to changes in the surface hydrophobicity in Pat-1 and its double mutant. This domain opening and increasing solvent exposure of the inner core of the VL domains occurs first in the lag phase, followed by changes in secondary structure as detected by CD spectroscopy (Figs. 3a, 4b; Supplementary Fig. 8c).

To extend our analyses of conformational rearrangements via the accessibility of tryptophan, fluorescence quenching by acrylamide was assessed. The quenching is stronger the more solvent accessible the tryptophan residue is. There is a steep increase in fluorescence quenching already at the beginning of the lag phase and the amplitude continues to grow up to 1 h. This indicates that the structural rearrangement we observed via changes in tryptophan fluorescence involves the rapid repositioning of the buried tryptophan to a solvent-exposed position (Fig. 4c).

**Fig. 5:**
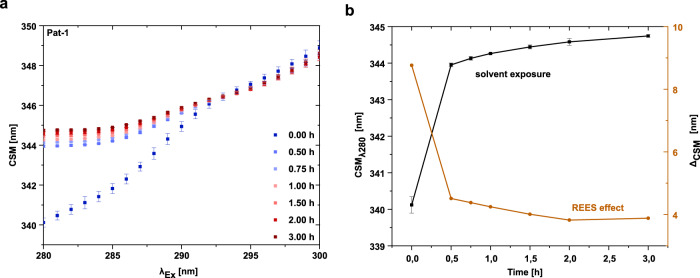
The REES effect of Pat-1 V_L_ during the lag phase. **a** Change in the CSM for the corresponding excitation wavelengths from 280 to 300 nm. **b** Conclusion of the REES effect (orange) and solvent exposure (black) at the different timepoints. Measurements were carried out in triplicates. Error bars respresent the respective SEM of the triplicates.

To obtain further structural insight in the changes of the tryptophan environment, we employed red edge excitation shift (REES) spectroscopy^33^. The REES effect is driven by the dipole interactions of the fluorophore with its surrounding: a rigid or a completely solvent-exposed surrounding leads to a smaller effect than a flexible protein present in different conformational states^34^. For the Pat-1 V_L_ domain, we observed an increase of the center of spectral mass (CSM) after 0.25 h compared to the start of the reaction (Fig. 5a, b). This implies a strong increase in solvent exposure of the tryptophan at the beginning of the lag phase due to a domain opening. After 0.25 h, a second, slower phase became apparent, which increased over time (Fig. 5B). The higher REES effect suggests a rugged free energy landscape of the V_L_ domain at the beginning of the reaction, which decreases quickly, indicating that there are less conformational states available, which influence tryptophan fluorescence relaxation. The smaller REES effect is not due to an extensive unfolding of the protein, as in the presence of 6 M urea a pronounced shift to smaller values for the REES effect was observed (Supplementary Fig. 9c). Pat-1 L15P L82Q shows a similar effect concerning increased solvent exposure and decreasing REES effect (Supplementary Fig. 9c). The later stays the same for the germline while also an increase in the solvent exposure can be observed matching the fluorescence data (Supplementary Figs. 8b, 9b). Thus, the two phases of the REES kinetics during the lag phase may be related to the initial partial unfolding with a concomitant higher solvent accessibility or a higher rigidity due to dimerization followed by oligomer formation and rearrangement in the oligomer. In contrast to the fibril forming proteins, the germline accesses a lower amount of conformational states as indicated by the overall lower REES effect (Supplementary Fig. 9c)

## Discussion

The conformational switch from the native to the fibrillary state in amyloidosis comprises a lag phase, including a primary nucleation step, a transition phase in which fibrils start to form and elongate, and a final plateau phase in which fibrils are present and an equilibrium is reached^16,35^. The same general scheme was assumed to apply for AL amyloidosis^6,36–38^. However, the molecular events occurring in early phases of the fibrillation process were largely unknown. In this study, we determined the conformational transitions and the molecular species formed in the lag phase that predispose the pathogenic V_L_ domain to fibril formation.

We show that, starting from the monomeric patient LC truncation Pat-1^6^, small amounts of dimers are formed in the first hour of the lag phase. They might be caused by a dynamic equilibrium between these two states. Later on, the peak shoulder is not readily visible, however, we presume that a fraction of dimers is still present. As the dimeric fraction is the first observable step that differs between native and fibril-inducing conditions, these dimers reflect conformational changes which lead to destabilized monomers which finally form fibrils^6^. Of note, we consider these dimers as non-native species induced by the increasing hydrophobic surface at a beginning unfolding. They need to be differentiated from the native dimers observed for a number of LCs. In this context, the dimers either do not interfere with fibril formation^36,39^ or they may exert a protective role against fibrillization^40–42^. In addition, after 0.25 h of the lag phase, a specific pattern of higher oligomers was detected which rearranged before the assembly into fibrils starts. Concomitantly, the monomers decrease during the lag phase. The oligomer fraction shows a complex behavior that slightly differs between AUC and TEM analysis. According to AUC analysis, the amount of oligomers first increases and then decreases until they completely disappear when fibrils are formed. This hints at the formation of a nucleus prior to fibril formation as proposed for other amyloid reactions^43,44^. The cooperative transition to the fibrillary state supports the view that a specific conformational state of the oligomers is the seed for polymerization. Accordingly, the oligomeric species were always present in lower amounts compared to soluble monomer/dimer fraction or insoluble fibrils as described for other amyloids^16^. TEM micrographs of Pat-1 showed oligomers that could correspond to hexamers, which increase during the lag phase in number but not in size; the Pat-1 double mutant L15P L82Q formed similar oligomers rapidly before fibril formation. As negative staining involves the incubation in a low pH uranyl acetate solution, we assume that this dissolves the higher oligomers. Thus, the hexamer is the most stable oligomeric species. In contrast to Pat-1, the WT-1 oligomer species exhibited clustering and unspecific aggregation in TEM analysis.

According to the known secondary and tertiary structure of the monomers as well as the fibrils, major conformational rearrangements have to occur in V_L_ prior to fibril formation as the domain consists of a two-layer sandwich structure composed of antiparallel β-strands, whereas the amyloid fibrils in AL amyloidosis exhibit a cross-β sheet topology consisting of parallel β-sheets^14,15,45,46^. The single tryptophan residue buried in the core of the domain is an excellent spectroscopic probe for conformational changes as it reports on variations in its local environment^45^. Analysis of the intrinsic tryptophan fluorescence revealed that the microenvironment of the tryptophan residue in the core starts to change already in the very beginning of the lag phase as seen by an increase of overall fluorescence intensity. This effect increases with time indicating that the domain structure increasingly changes. The distancing of the tryptophan residue from the disulfide bond and the enhanced accessibility reflects the opening of the β-barrel structure while the overall secondary structure elements still remain largely intact as judged from the FUV CD measurements. This domain opening goes along with the formation of small amounts of dimers as seen in the AUC sedimentation profiles. Thus, we hypothesize that even though the overall secondary structure of the V_L_ monomer does not change during initial oligomerization, a state appears in which the packing starts to rearrange, and hydrophobic residues become surface-exposed. ANS binding experiments show an early increase of surface-exposed hydrophobicity, which seems to coincide with domain opening and oligomer formation. At this step, a further conformational reorganization occurs that fosters oligomerization via hydrophobic interactions, as observed for other amyloidogenic proteins^47–49^. The acrylamide quenching experiments give additional support to the idea of conformational rearrangements during oligomer formation. It monitors the accessibility of a quencher to a fluorophore and thus is a marker for solvent accessibility. The increase of the quenching constant K from the beginning of the lag phase onwards implies a higher solvent exposure of the buried tryptophan along with the partial unfolding of the V_L_ domain. REES experiments further revealed that an increased solvent accessibility of the tryptophan residue, monitored by the CSM at an excitation wavelength of 280 nm, takes place. This again supports the notion of domain opening concluded from changes in tryptophan fluorescence and ANS binding. The increase of CSM values at the y-intercept goes along with a decrease in the REES effect. The emission spectrum of a fluorophore is highly dependent on its environment since it is influenced by the dipole interaction with water. A more solvent-exposed surrounding thereby leads to a smaller REES effect, as shown by the unfolding of Pat-1 with urea. The magnitude of the REES effect can provide information about the free energy landscape of a protein^34^. A higher REES effect is based on a higher number of discrete conformational states as seen for Pat-1 and Pat-1 L15P L82Q. Upon oligomerization this REES effect decreases, which also implies a decrease in conformational states. In contrast, WT-1 shows unfolding events but the accessible conformational states do not increase. Since the fluorescence intensity does not decrease during the lag phase and during fibril formation, the tryptophan does not shift back to its position in close proximity of the quenching disulfide bond. These results are in excellent agreement with the cryo-EM structure of AL fibrils, where the conserved tryptophan residue is found close to the fibril surface. Furthermore, the disulfide bond is intact but the interactions of residues including the tryptophan differ significantly from that in the native protein^14,50^. We assume that after initial unfolding events and partial dimer formation from folded monomers, a subsequent fast association of monomeric or dimeric species to oligomers occurs. Preceding fibril formation but after the initial unfolding events, a shift in the FUV CD minima became visible which we assume represents the rearrangement of the β-sheets. The change of local minima fits to the switch of antiparallel to parallel β-sheet fold^26^. Thus, the CD results indicate that this timepoint marks further important structural rearrangements induced by intermolecular interactions. The rapid reaction into fibrillary structures with features of a supramolecular β-sheet formation reveals that the rearranged species is transient, meta-stable and thus potentially presents the nucleus for fibril formation. The β-sheet formation in fibrils was also observed in FTIR, the rapid switch after ~2 h could not be resolved in detail. However a difference in the amide II region emerged. Of note, the FTIR measurements required extensive sample processing and could not be conducted in a time-resolved manner similar to the CD. In this context, it should be noticed that in general conditions were kept identical between the different methods used. However, additives like ANS or ThT, or slightly different conditions like in the AUC experiments might potentially impact the conformational equilibrium. However, the methodologies have been well established for the addressed questions and the results obtained are in line with each other, supporting the individual evidence.

Compared to the disease-related V_L_ domains, the germline behaves differently in important aspects. While it also undergoes partial unfolding that leads to an increase in the tryptophan fluorescence and a slight increase in surface hydrophobicity, the conformational state of the germline remains constant during the lag phase and there are no observable changes in the secondary structure. The oligomeric species formed end in amorphous aggregates.

**Fig. 6:**
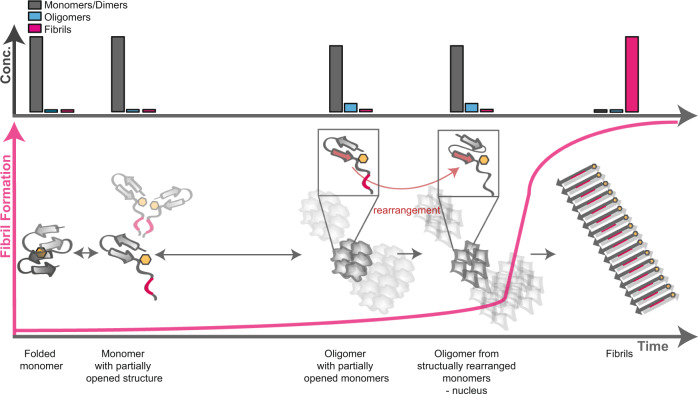
Model of the events taking place in the lag phase of fibril formation. Folded V_L_ monomers undergo a partial structural opening whereupon they assemble to dimers, and oligomers (hexamers and multiples of hexamers). Prior to fibril formation, the monomers in these oligomers structurally rearrange resulting in parallel β-sheets. This variant is the nucleus of fibrillization. The above bars show the concentration of each species at the respective timepoint of the fibril formation process. The concentration of oligomers is low at all timepoints.

In summary, our biophysical analyses reveal a multi-step conformational transition from a folded monomeric β-barrel domain into an amyloid fibril (Fig. 6). It starts with an initial domain opening and partial unfolding in which the β-strands of the V_L_ monomers are preserved. The increase in surface hydrophobicity fosters dimerization and concomitant assembly into hexamers and multiples of hexamers. In this process further rearrangements occur, which reduce conformational flexibility. Consistent with the idea that the oligomers are the species with the highest Gibbs free energy, they are present only transiently and at low concentration. It was previously reported that the life time of oligomers of amyloid proteins can vary significantly. Also, oligomers may have a higher tendency for dissociation to monomers than for fibril formation^51^.

In the context of our study, it is useful to compare the concept established for AL amyloidosis with other fibril forming proteins. The dialysis-related amyloid precursor β_2_-microglobulin also consists of an immunoglobulin fold. It was shown that starting from the natively folded monomer, an unfolded state emerges and subsequent oligomerization precedes fibril formation^39,52–54^. Clustering following unfolding has also been suggested for amyloidosis involving α-helical precursors^55^. Our study shows that the decisive committing step for the amyloid pathway occurs in the context of the oligomer. In contrast, unfolding and initial oligomerization were also observed for the nonamyloidogenic germline protein. However, this protein subsequently ends up in amorphous aggregates. Thus, it seems that only once the oligomers undergo a critical structural rearrangement nuclei are formed, which are rapidly transformed into amyloid fibrils. For further insights into the structure of the oligomers, high resolution methods like cryo-EM or solid state NMR need to be applied in the future. Especially, since pathogenic effects could be ascribed not only to insoluble fibrils but also to oligomers^56^. Furthermore, a molecular understanding of the conformational transitions in the lag phase of different amyloidoses may result in the emergence of general concepts across diseases.

## Methods

All chemicals were from Merck (Darmstadt, Germany) or Sigma (St. Louis, USA). All measurements were carried out in PBS buffer containing small amounts of SDS^24,57,58^ (10 mM Na_2_HPO_4_ × 2 H_2_O; 1.8 mM KH_2_PO_4_; 2.7 mM KCl; 137 mM NaCl; 0.5 mM SDS) at pH 7.4 and 37 °C, unless otherwise stated. Data were analyzed using Origin 2019.

### Expression and purification of Pat-1, Pat-1 L15P L82Q, and WT-1

The origin of the Pat-1 sequence and the recombinant expression and purification of Pat-1, Pat-1 L15P L82Q, and WT-1 was described before. Also, the generation of the point mutations with the pirmers TAGCGGTAGCCCGGGTCAGAGCATTA (+) and ACGCTTGCAGGCTGGGTC (−) has been previously described. In brief, the plasmids were transformed in E. coli BL21 (DE3)-star cells and protein expression took place at 37 °C overnight. Cells were harvested and inclusion bodies were prepared. The pellet was solubilized and unfolded in 25 mM Tris-HCl (pH 8), 5 mM EDTA, 8 M urea, and 2 mM β-mercaptoethanol at room temperature for a minimum of 2 h. Afterwards, the protein was loaded onto a Q-Sepharose anion exchange column equilibrated in 25 mM Tris-HCl (pH 8.0), 5 mM EDTA and 5 M urea. The LCs and V_L_s were eluted in the flow-through fractions and refolded by dialysis against 250 mM Tris-HCl (pH 8.0), 100 mM L-Arg, 5 mM EDTA, 1 mM oxidized glutathione and 0.5 mM reduced glutathione at 4 °C overnight. To remove aggregates and impurities, the refolded proteins were purified using a Superdex 75 16/60 gel-filtration column (GE Healthcare, Uppsala, Sweden) equilibrated in PBS buffer. Recovery and purity of intact proteins were analyzed by SDS-PAGE.

### Sample preparation for lag phase analysis

For studying the different timepoints, for all measurements unless otherwise stated, 15 µM of Pat-1 were prepared in the prior described assay buffer in a 1.5 mL Eppendorf reaction tube and were incubated at 37 °C ± 2 and 750 rpm shaking in a Thermomixer compact (Eppendorf, Hamburg, Germany). For 0 h timepoints, the sample was put on 37 °C and removed before shaking. All other timepoints were removed after the corresponding time shaking. All samples were kept on ice afterwards. To confirm the interruption of fibril formation at the different timepoints the shaking was stopped and the samples were kept on ice or at 25 °C for 3 h prior to ThT fluorescence analysis.

### Analytical ultracentrifugation (AUC)

AUC measurements were carried out using an Optima AUC (Beckman, Krefeld, Germany) equipped with absorbance optics. The protein concentration for the measurements was 30 µM due to a low data resolution at lower concentrations. A total volume of 350 µL per sample was loaded into assembled cells with quartz windows and 12 mm-path-length charcoal-filled epon double-sector centerpieces. The measurements were performed at 42,000 rpm in an eight-hole Beckman-Coulter AN50-ti rotor at 20 °C. Sedimentation was continuously scanned with a radial resolution of 10 µm and monitored at 280 nm. Data analysis was carried out with software SEDFIT using the continuous c(S) distribution mode^59,60^.

### Far-UV (FUV) circular dichroism (CD) measurements

For Pat-1, FUV CD spectra were recorded from 197–260 nm using a Chirascan-plus CD spectrometer (Applied Photophysics, Leatherhead, England). Measurements were recorded with a bandwidth of 1.0 nm in 1.0 nm steps and 0.5 s time per point at a temperature of 37 °C. All measurements were performed using a 15 µM protein solution in a quartz cuvette with 1 mm pathlength. The spectra show an average of 10 individual measurements.

For Pat-1 L15P L82Q and WT-1 FUV CD spectra were recorded from 197 to 260 nm using a Jasco J-1500 (JASCO, Pfungstadt, Germany). Measurements were recorded with a data pitch of 0.1 nm and a scanning rate of 20 nm/min time per point at a temperature of 37 °C. All measurements were performed using a 15 µM protein solution in a quartz cuvette with 0.5 mm pathlength. The spectra show an average of 20 individual measurements.

### Attenuated total reflection Fourier transform infrared spectroscopy (ATR-FTIR)

After the respective timepoint of the lag phase, samples were dialyzed in 10 mM Na_2_HPO_4_ × 2 H_2_O; 1.8 mM KH_2_PO_4_ overnight and 250 µl were dried on one side of a germanium crystal under nitrogen flow. The crystal was mounted into a home-made, gas-tight holder and the latter in the FTIR spectrometer (VERTEX 70 from Bruker, Germany). The samples were equilibrated in the measurement chamber under nitrogen flow for 30 min and subsequently incubated with D_2_O saturated nitrogen flow for H/D exchange of bound water fractions. The kinetics of H/D exchange was repeatedly recorded with 32 scans at 2 cm^−1^ resolution until the spectra remained unchanged. After 50 min the final spectra were recorded, accumulating 1024 scans. The linear baseline was subtracted from spectra for further analyses and documentation.

### 8-Anilino-1-naphtalenesulfonic acid (ANS) and Tryptophan Fluorescence

For measurement of ANS binding, samples were taken at different timepoints and incubated with 150 µM ANS for 1 h. Spectra were recorded from 400 to 650 nm with an excitation of 380 nm. For intrinsic Tryptophan fluorescence measurements spectra were recorded from 300–450 nm with an excitation wavelength of 280 nm. All fluorescence measurements were carried out using a Jasco FP-8500 Spectrofluorometer (JASCO, Pfungstadt, Germany) at 25 °C. The settings included excitation and emission bandwidth of 5 nm each, 4 s response time, a data interval of 1 nm and 200 nm/min scan time.. For Pat-1 L15P L82Q and WT-1 spectra were recorded at a Tecan Infinite 200 PRO M Nano^+^ with a data interval of 1 nm and an amplification of 100. Depicted spectra show the average of three individual measurements. Samples were taken from a 15 µM protein solution incubated at 37 °C and 700 rpm.

### Acrylamide quenching

For Acrylamide quenching, samples were taken at different timepoints and 0 M to 1 M acrylamide in 1 × PBS in 0.2 M steps were added to 5 µM of the protein. The tryptophan fluorescence was recorded from 300 to 400 nm with an excitation of 280 nm. The measurement was carried out at 37 °C at a Tecan Infinite 200 PRO M Nano^+^ with an amplification of 157 and a data interval of 1 nm. The Stern-Volmer quotient F_0_/F was calculated with the maximal fluorescence intensity at 332 nm, while F_0_ is the value at 0 M and F the fluorescence intensity at the respective acrylamide concentration^61,62^. The raw data was linearly fitted. The slope represents the quenching constant regarding Eq. (1):

1 $$\frac{{F}_{0}}{F}=1+K\left|Q\right|$$

### Red edge excitation shift (REES)

The REES effect at different timepoints was conducted at a Jasco FP-8500 Spectrofluorometer at 25 °C with a scanning rate of 500 nm/min. The setup included a bandwidth of 5 nm for excitation and emission, a response time of 2 s and an interval of 1 nm. The tryptophan emission was monitored from 315 to 400 nm with an excitation scan from 280 to 300 nm. For data analysis, the center of spectral mass (CSM) was calculated as reported previously^34^.

### Thioflavin T (ThT) assay

Fast fibril formation assays followed by ThT fluorescence were performed in 1.5 mL Eppendorf tubes. For Pat-1, kinetics were followed by measuring at different timepoints in a 10 × 2 mm quartz cuvette using a FluoroMax-4 spectrofluorometer (Horiba Jobin Yvon, Bensheim, Germany) and a Jasco FP-8500 Fluorescence Spectrometer (JASCO, Pfungstadt, Germany). Kinetics were followed by measuring at 440 nm excitation and 480 nm emission with slit widths of 3 nm or 2.5 nm for excitation and 4 nm or 5 nm for emission, respectively. For Pat-1 L15P L82Q and WT-1, kinetics were followed by measuring at different timepoints in a 96-well UV star plate (Greiner Bio-One, Frickenhausen, Germany) at a Tecan Infinite 200 PRO M Nano^+^ with an amplification of 60 and a data interval of 1 nm, The ThT fluorescence was measured from 460 to 500 nm with an excitation wavelength of 440 nm. The data were plotted by using the ThT fluorescence at 480 nm. To remove oligomeric species and aggregated protein and prevent seed formation, monomer isolation was performed prior to the experiment by ultracentrifugation in an Optima^TM^ MAX-E ultracentrifuge, (Beckman, Krefeld, Germany).

### Sodium dodecyl sulfate polyacrylamide gel electrophoresis (SDS-PAGE)

SDS Gels were cast in two steps pouring first the 18% separating gel solution and then the 5% stacking gel solution. For sample preparation, samples taken from 15 µM Pat-1 solution at different timepoints were centrifuged and separated into supernatant and pellet fraction. The pellet was washed twice with PBS. Supernatant samples were diluted with 5 × reducing Laemmli buffer, pellets were dissolved in 1 × reducing Laemmli buffer. Both were heated at 95 °C for 5 min for denaturation. 20 µL of the respective samples were applied into the gel pockets. The gel was run at 30 mA for 40 min and stained with Fairbanks A (25% Isopropanol, 10% Acetic Acid, 0.05% Coomassie R) and destained with Fairbanks D (10% Acetic Acid) afterwards.

### Transmission electron microscopy (TEM)

TEM samples were prepared by pipetting 10 µL samples onto a 200-mesh activated copper grid and incubating for 1 min. The samples were washed with 2 × 10 µL H_2_O and negatively stained with 8 µl of a 1.5% (w/v) uranyl acetate solution for 1 min. Excess solutions were removed with a filter paper. TEM micrographs were recorded on a JEOL JEM-1400 Plus transmission electron microscope (JEOL Germany GmbH, Freising, Germany) at 120 kV.

### Statistics and reproducibility

All representative TEM micrographs in this publication have been chosen from 3 to 20 images taken from a negative stained 200-mesh activated copper grid.

### Reporting summary

Further information on research design is available in the Nature Research Reporting Summary linked to this article.

## Supplementary information

Supplementary Information

Reporting summary

## Data Availability

All data generated during this study are available in the article and supplementary information or from the corresponding author. Source data are provided with this paper.

## Source data

Source Data
