# Supplementary material for: Dissection of the amyloid formation pathway in AL amyloidosis
Source: Nat Commun. 2021 Nov 11;12:6516. doi: 10.1038/s41467-021-26845-0 (PMC8585945; doi:10.1038/s41467-021-26845-0)
Supplement: Supplementary file 1 — Supplementary Information [file 41467_2021_26845_MOESM1_ESM.pdf]

Supplementary information

Dissection of the amyloid formation pathway in AL amyloidosis

Pamina Kazman[1,#], Ramona M Absmeier[1,#], Harald Engelhardt[2] , Johannes Buchner[1*]

[1] Department Chemie, Technische Universität München, 85748 Garching, Germany

[2] Department Molecular Structural Biology, Max-Planck-Institute of Biochemistry, 82152 Martinsried, Germany

# contributed equally

* corresponding author: johannes.buchner@tum.de

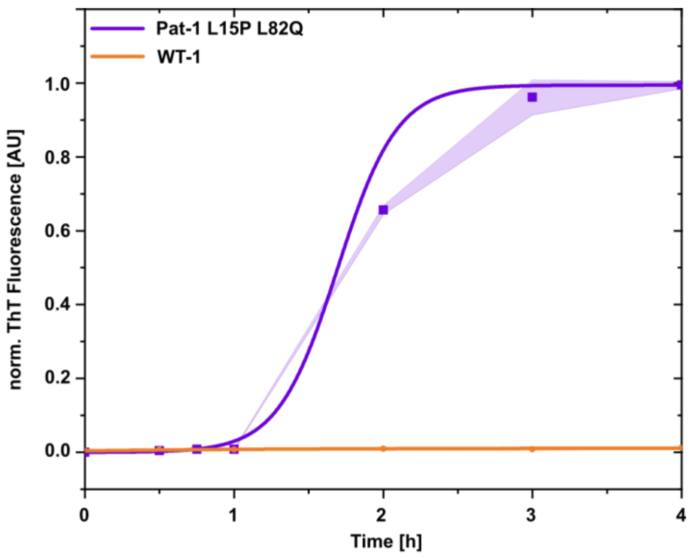

Supplementary Figure 1. Fibril formation of Pat-1 L15P L82Q (violet) and the germline WT-1 (orange). Shades represent the SEM of n=3.

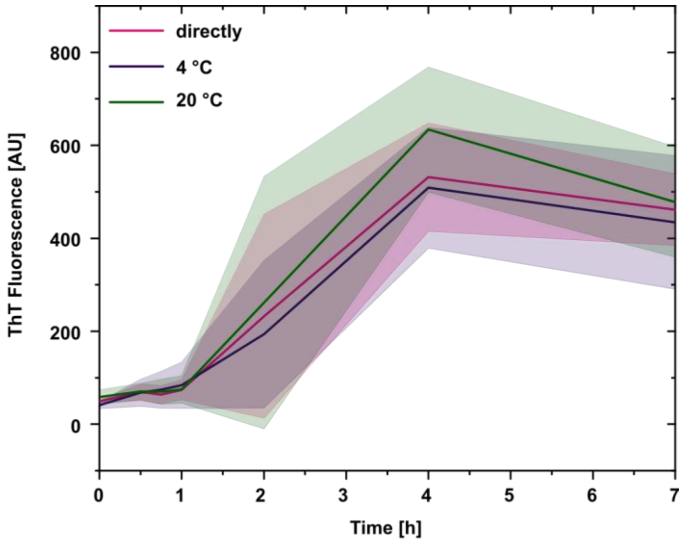

Supplementary Figure 2. Fibril formation stop. The inhibition of further fibril formation when shaking was monitored by ThT fluorescence when directly measuring the samples after stop shaking and measuring them after leaving them at either 4 °C or 20 °C for 3 hours. Shades represent the SEM of n=3.

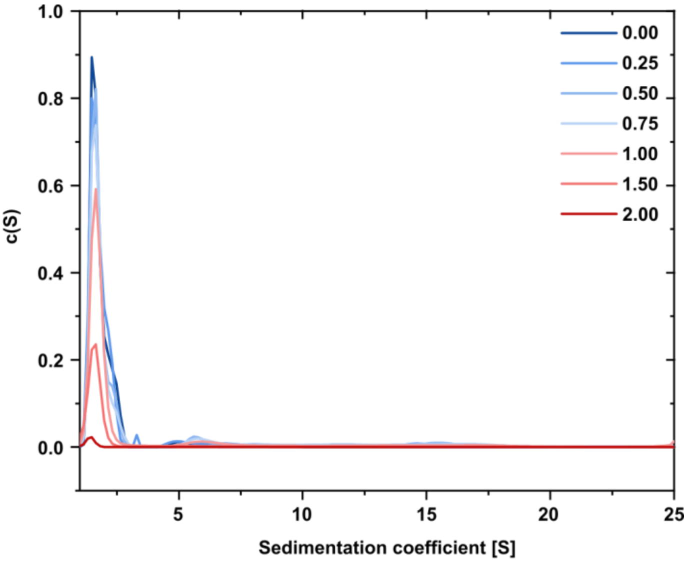

Supplementary Figure 3. Full sedimentation profile of oligomeric species present in the lag phase. AUC data show the sedimentation profiles over time as analyzed by the Sedfit software.

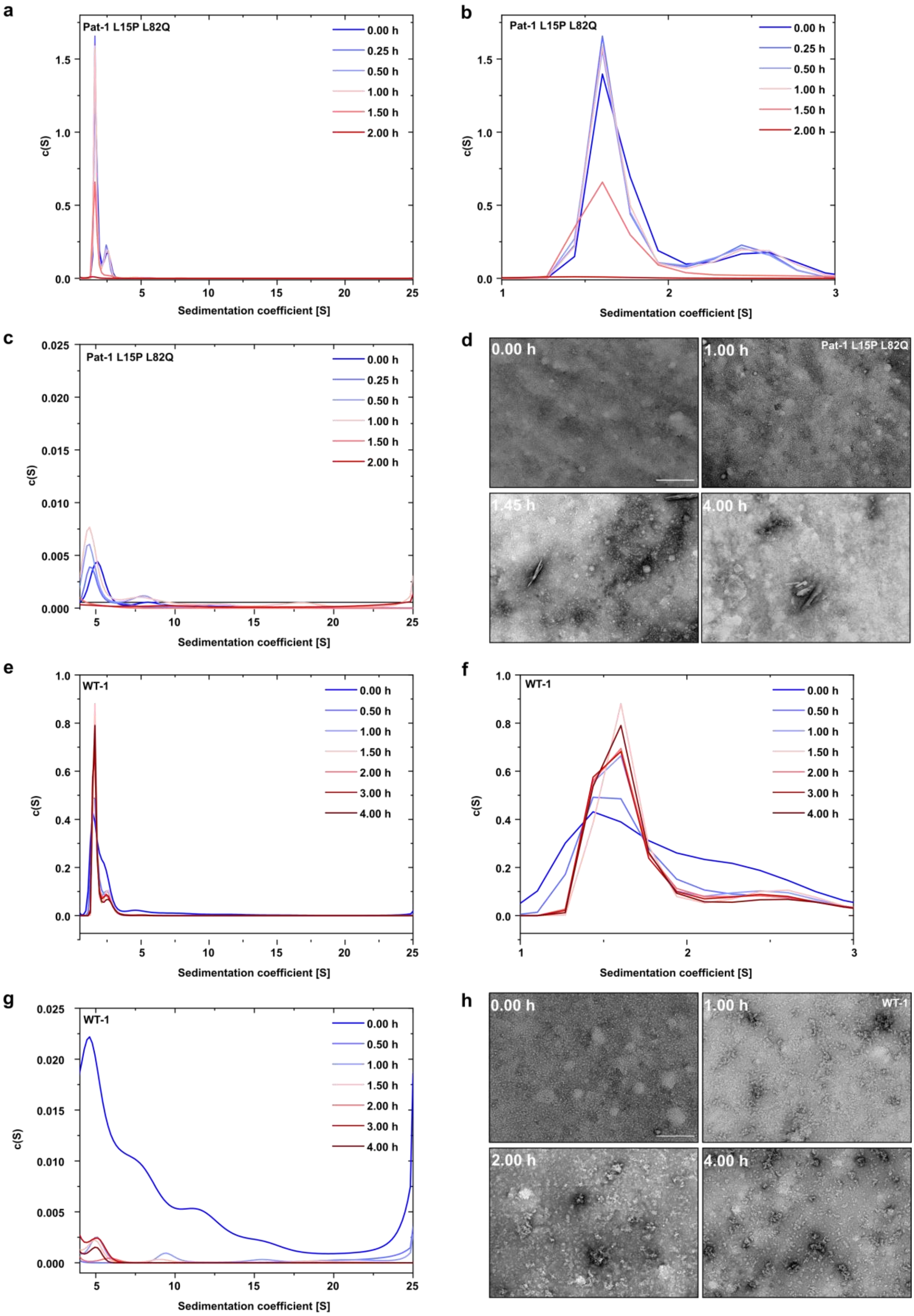

a
Pat-1 L15P L82Q
0.00 h
0.25 h
0.50 h
1.00 h
1.50 h
2.00 h
c(S)
Sedimentation coefficient [S]
b
Pat-1 L15P L82Q
c
Pat-1 L15P L82Q
d
0.00 h
1.00 h
Pat-1 L15P L82Q
1.45 h
4.00 h
e
WT-1
0.00 h
0.50 h
1.00 h
1.50 h
2.00 h
3.00 h
4.00 h
f
WT-1
g
WT-1
h
0.00 h
1.00 h
WT-1
2.00 h
4.00 h

Supplementary Figure 4. Analysis of oligomeric species of the Pat-1 mutant L15P L82Q and the germline WT-1. a) Full sedimentation profile of the lag phase of Pat-1 L15P L82Q analyzed by the Sedfit software b) monomer peak of the sedimentation profile of Pat-1 L15P L82Q c) oligomer peaks of the sedimentation profile of Pat-1 L15P L82Q d) TEM micrographs of the oligomeric and fibril species of Pat-1 L15P L82Q during the lag phase. e) Full sedimentation profile of the Lag phase of WT-1 analyzed by the Sedfit software f) monomer peak of the sedimentation profile of WT-1 g) oligomer peaks of the sedimentation profile of WT-1 h) TEM micrographs of the oligomeric species of WT-1 during the lag phase. Scale bars represent 200 nm.

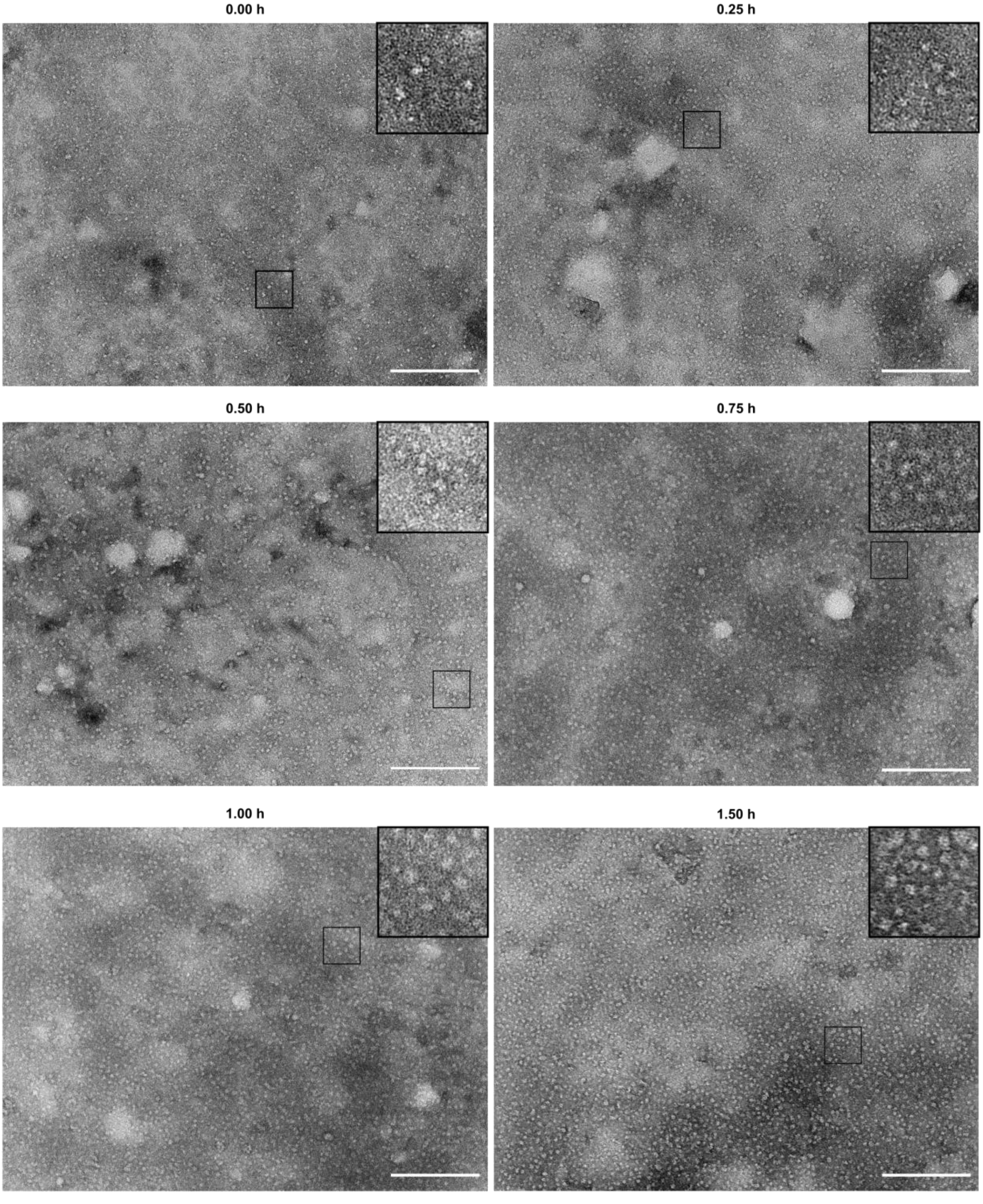

Supplementary Figure 5. TEM micrographs of oligomers at different time points during the lag phase. Area of black outlined squares is shown in 30x magnification. The scale bars represent 200 nm.

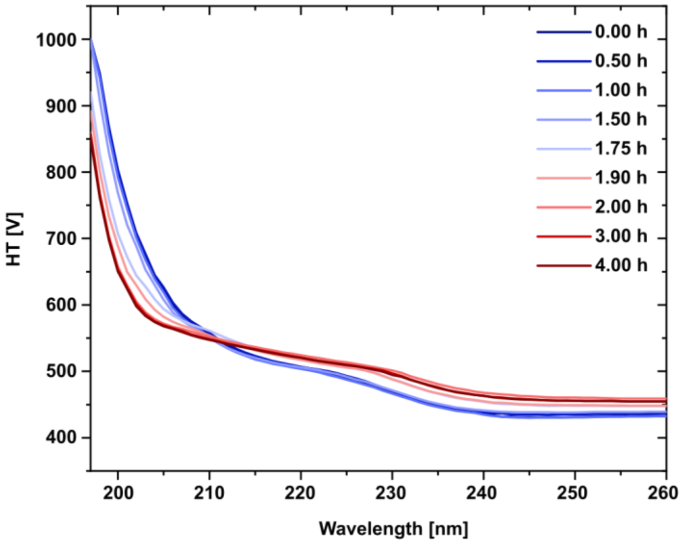

Supplementary Figure 6. HT signal of the CD spectrum of Pat-1 V_L.

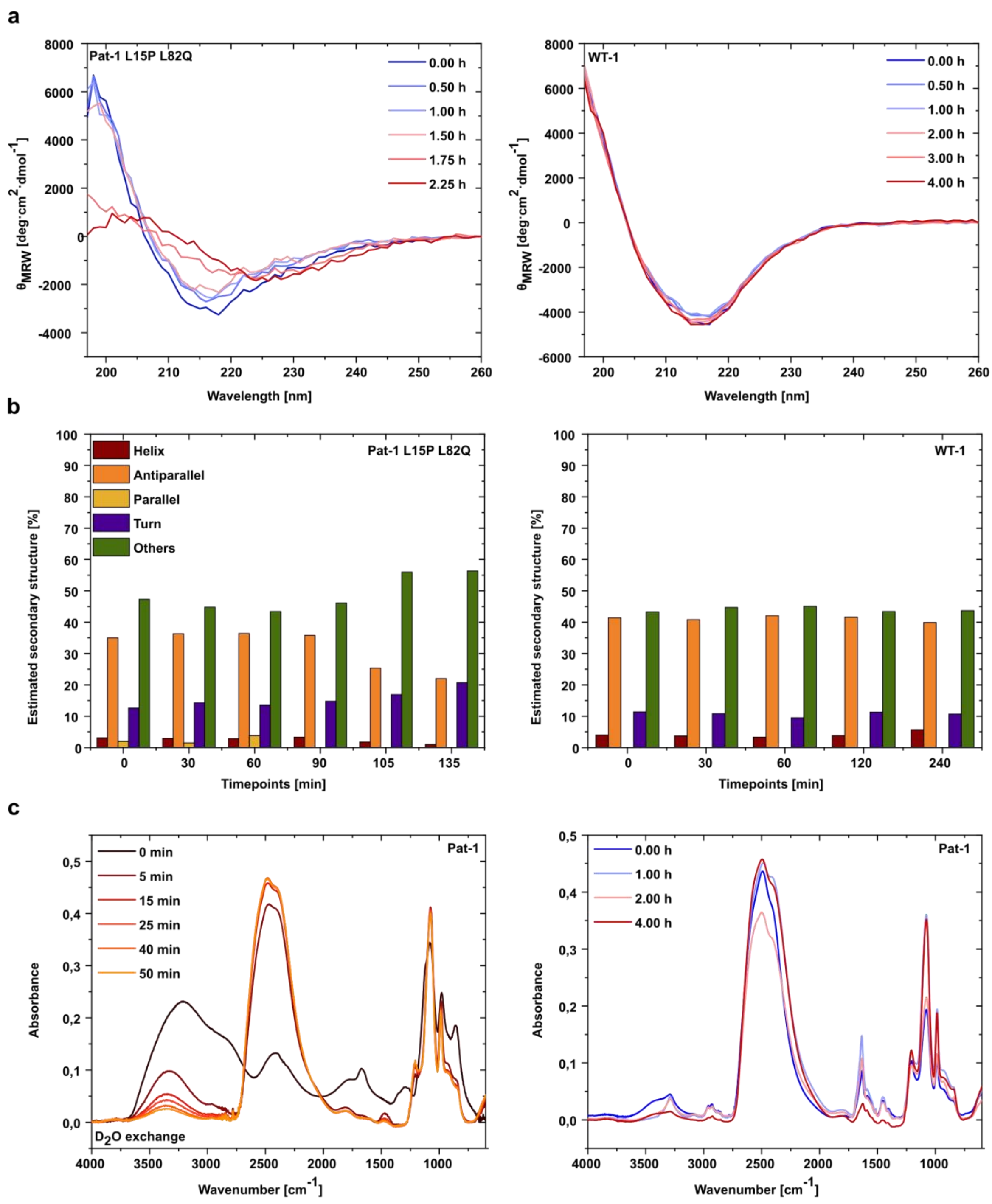

Supplementary Figure 7. Secondary structure analysis of Pat-1, WT-1 and Pat-1 L15P L82Q. a) CD spectra of different lag phase time points until fibrils formation of the double mutant L15P L82Q (left panel), and CD spectra of the germline WT-1 (right panel). b) Secondary structure analysis of the CD spectra of Pat-1, its double mutant and the germline with the BeStSel algorithm. c) ATR-FTIR analysis of Pat-1. The left panel shows the success of the D_2O exchange of the buffer background which is also representative for the exchange kinetics concerning samples including protein. The right panel shows the FTIR spectra of Pat-1 after 50 minutes of D_2O exchange.

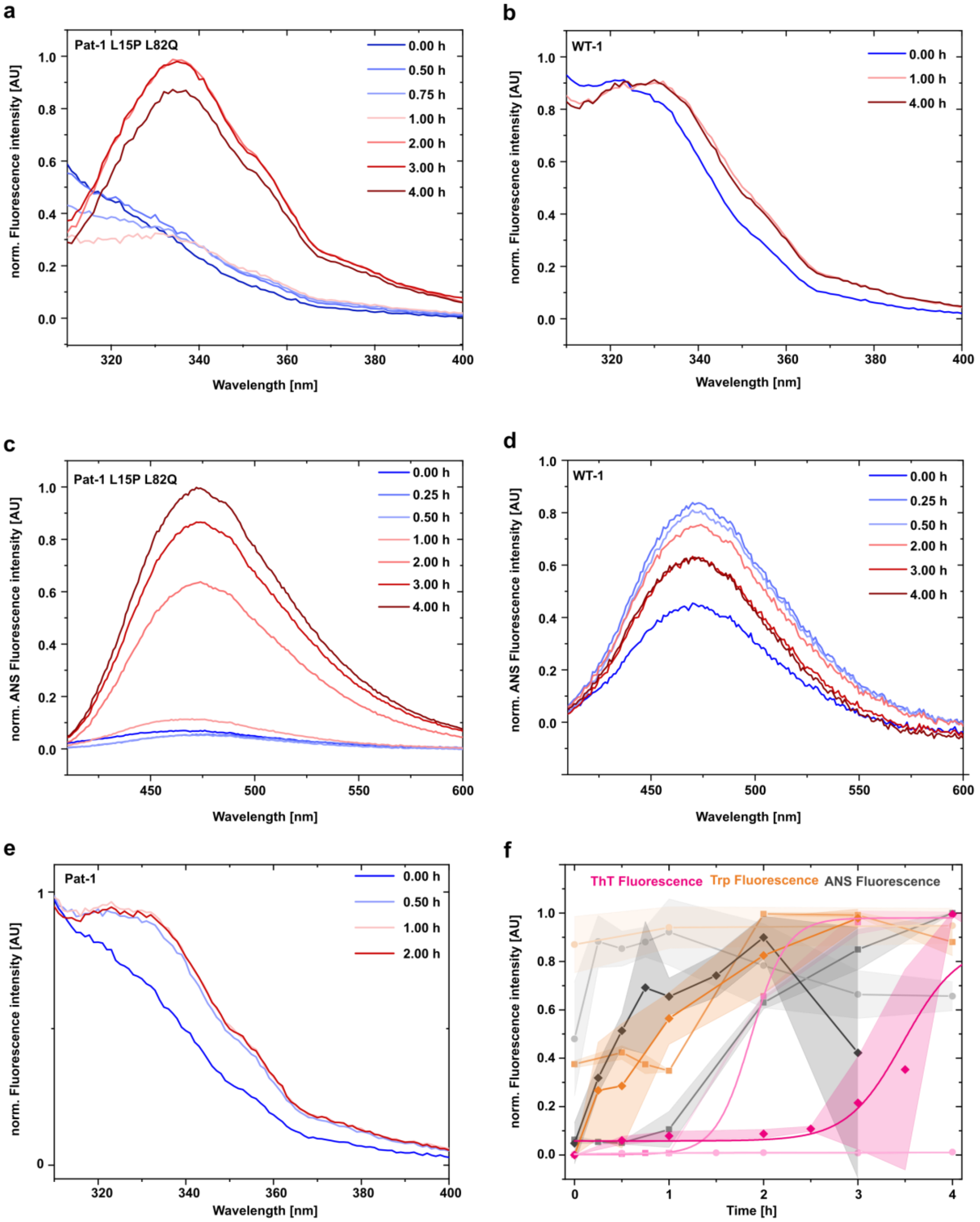

Supplementary Figure 8. Structural changes during Lag Phase of Pat-1 L15P L82Q and the germline WT-1 a) Trp Fluorescence spectra of Pat-1 L15P L82Q. b) Trp Fluorescnce changes of WT-1 during Lag phase. c) Changes of exposed hydrophobicity followed by ANS fluorescence of Pat-1 L15P L82Q. d) Changes of exposed hydrophobicity followed by ANS fluorescence of the germline WT-1. e) Fluorescence intensity chances of Pat-1 during the lag phase measured at 37 °C. f) Summary of the Fluorescence data of all constructs. The different proteins are depicted by a diamond (Pat-1), square (Pat-1 L15PL82Q) or a circle (WT-1). The colors of the symbols correspond to the method as indicated in the graph with different tones of the color used for the different proteins. Shades represent the SEM of n=3.

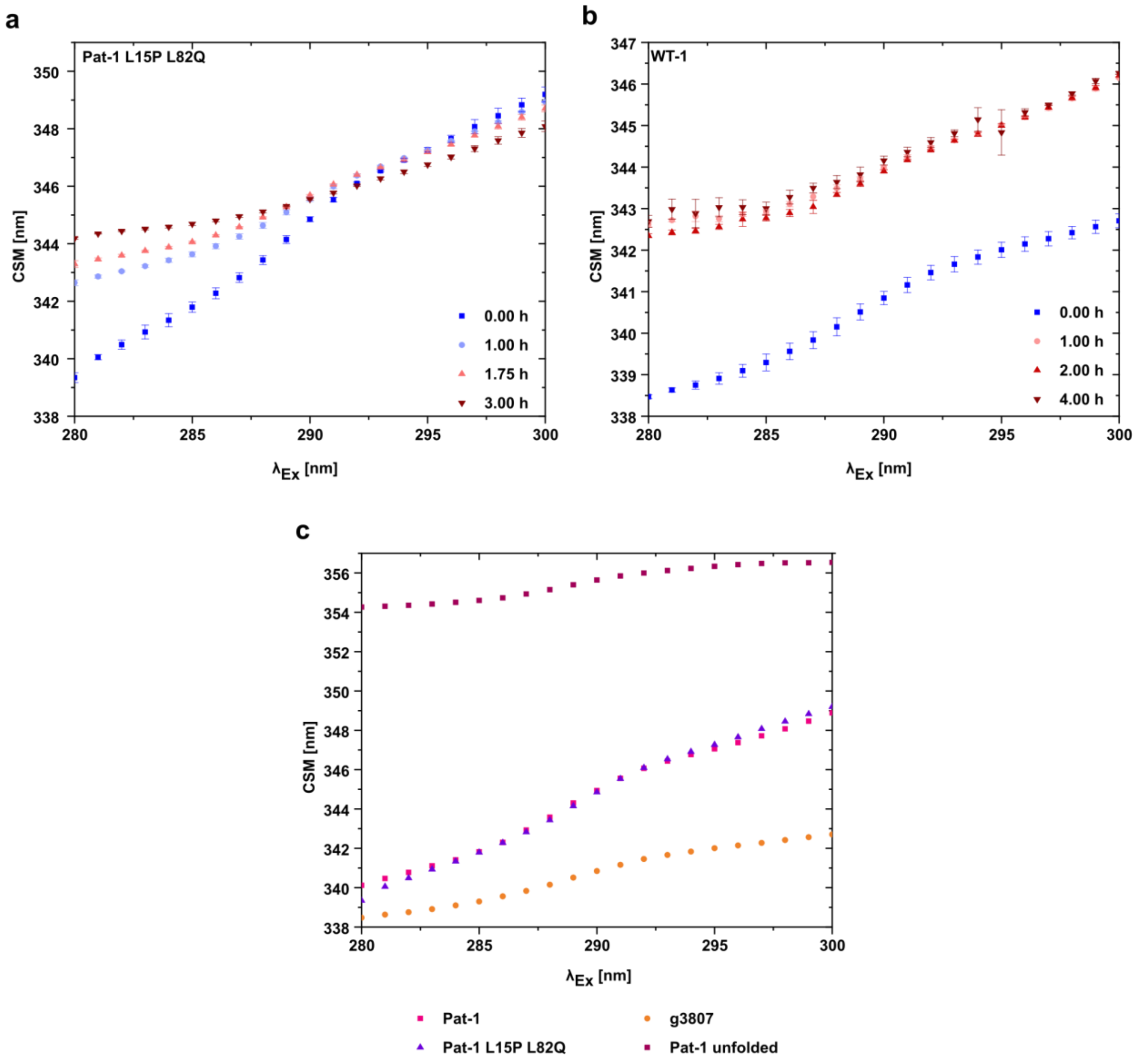

Supplementary Figure 9. The REES effect of Pat-1 L15P L82Q and WT-1 V_L during the lag phase. a) Change in the CSM for the corresponding excitation wavelengths from 280 to 300 nm of Pat-1 L15P L82Q V_L b) Change in the CSM for the corresponding excitation wavelengths from 280 to 300 nm of WT-1 (n=3). c) Comparison of the REES effects of Pat-1, Pat-1 L15P L82Q and WT-1 at 0h. Pat-1 VL in 6 M urea is shown as a control for an unfolded protein (n=3). Error bars represent SEM of n=3.

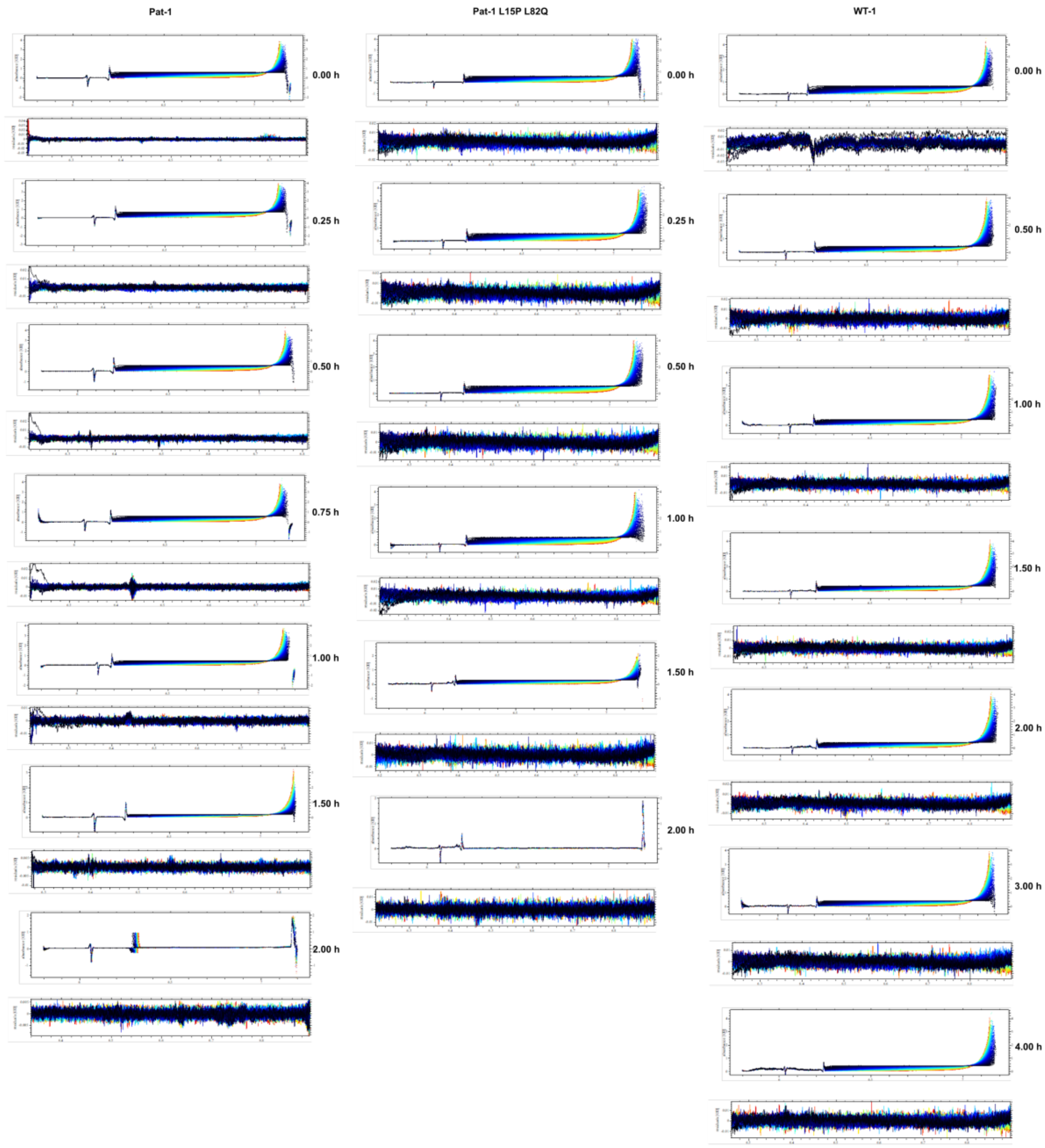

Supplementary Figure 10. AUC absorbance with residual raw data of Pat-1, Pat-1 L15P L82Q and WT-1.
